# Fatty Acid and Essential Oil Compositions of *Trifolium angustifolium* var. *angustifolium* with Antioxidant, Anticholinesterase and Antimicrobial Activities

**Published:** 2015

**Authors:** Abdulselam Ertaş, Mehmet Boğa, Nesrin Haşimi, Mustafa Abdullah Yılmaz

**Affiliations:** a *Department of Pharmacognosy, Faculty of Pharmacy, Dicle University, 21280 Diyarbakir, Turkey.*; b *Department of General and Analytical Chemistry, Faculty of Pharmacy, Istanbul University, 34116 Beyazıt, Istanbul, Turkey. *; c *Department of Nutrition and Dietetics, School of Health, Batman University, 72060, Batman, Turkey. *; d *Research and Application of Science and Technology Center (DÜBTAM), Dicle University, 21280 Diyarbakir, Turkey.*

**Keywords:** *Trifolium angustifolium* var. *angustifolium*, Fatty acid, Essential oil, Antioxidant, Antimicrobial, Anticholinesterase

## Abstract

This study represents the first report on the chemical composition and biological activity of *Trifolium angustifolium* var. *angustifolium*. The major components of the essential oil were identified as hexatriacontene (23.0%), arachidic acid (15.5%) and *α*-selinene (10.0%). The main constituents of the fatty acid obtained from the petroleum ether extract were identified as palmitic acid (29.8%), linoleic acid (18.6%) and oleic acid (10.5%). In particular, the water extract exhibited higher activity than *α*-tocopherol and BHT, which were used as standards in the ABTS cation radical scavenging assay and indicated higher inhibitory effect against acetylcholinesterase enzyme than the reference compound, galanthamine but exhibited weak activity in *β*-carotene bleaching, DPPH-free radical scavenging, and cupric-reducing antioxidant capacity assays. The petroleum ether extract exhibited higher activity than *α*-tocopherol which was used as standard in the *β*-carotene bleaching method at concentration 100 μg/mL. The acetone extract exhibited higher activity than *α*-tocopherol which was used as standard cupric reducing antioxidant capacity (CUPRAC) method at 100 μg/mL concentration. The acetone and methanol extracts were active on all microorganisms tested with a small zone diameter indicating weak activity.

## Introduction

There are 103 species of *Trifolium *(*Leguminosae*) genus in Turkey (1). Being a center of variety, Trakya (European Turkey) has 67 *Trifolium* taxa (2). In Turkish traditional medicine, some *Trifolium *species such as *Trifolium repens *Lin., *Trifolium arvense *Lin., *Trifolium pratense *Lin. are used as cough syrup, anesthetic, disinfectant and tonic (3). Additionally, some of the species are important feeding feedstuff for sheep and cattle in the Mediterranean region (4, 5). In previous scientific studies triterpene saponins (6), megastigmane glycosides (7), flavonoids (5, 8), chalcanol glucosides (9), steroids, phytylesters and lipids (10) have beeen isolated from *Trifolium *species.

It is believed that reactive oxygen species (ROS) and free radicals cause DNA strand breaks and damages the proteins and the cell membrane (11). As is known, the oxygen-centered radicals cause oxidative stress and that is the primary factor for a number of degenerative diseases. Epidemiological studies suggest that adding fruits, vegetables and cereals to your diet is essential to prevent chronic diseases such as cardiovascular diseases and some types of cancer (11). Hence, screening of the plant species to identify new antioxidants have become critically important in recent years. 

**A **literature survey showed that there are no phytochemical or biological reports on *T. angustifolium* var. *angustifolium*. Hence, aim of our study was to determine fatty acid and essential oil compositions, antioxidant, antialzheimer and antimicrobial activities of the petroleum ether, acetone, methanol and water extracts of *T. angustifolium* var. *angustifolium* growing in Turkey. The essential oil and fatty acid of *T. angustifolium* var. *angustifolium* was analyzed to determine its composition by GC/MS. This study is the first biological and phytochemical report on *T. angustifolium* var. *angustifolium*.

## Experimental

*General experimental procedures*

A Thermo pH-meter, a BioTek Power Wave XS, an Elma S15 ultrasonic bath and a vortex (LMS Co. LTD) were used for the activity assays. Ethanol, hexane, diethyl ether, chloroform, toluene, dichloromethane, methanol, potassium acetate, sulphuric acid, aluminium nitrate nonahydrate, aluminium chloride, ABTS, sodium acetate, nutrient broth, boric acid, nutrient agar, butylated hydroxytoluene were purchased from Merck (Germany), DPPH,  -carotene, H_2_O_2_, quercetin, pyrocathecol, acetic acid, sodium methoxide, Tween 40, DTNB, copper (II) chloride dihydrate (CuCl_2_.2H_2_O), linoleic acid, neocuproine, EDTA, acetylcholinesterase, butyrylcholinesterase from Sigma (Germany),  -tocopherol, acetylthiocholine iodide from Aldrich (Germany), galanthamine hydrobromide from Sigma-Aldrich (Germany), BHT from Fluka (Germany), sterile blank disc and antbiotic disc from Oxoid (United Kingdom), petroleum ether, sodium dihydrogen phosphate, sodium carbonate, sodium hydrogen phosphate, ammonium acetate from Reidel de Haen (Germany).

*Plant material*

A whole plant of *Trifolium angustifolium* L. var. *angustifolium* L. was collected from western Turkey (Istanbul)) in April 2012 by Dr. Abdulselam Ertaş and identified by Dr. Mine Koçyiğit (Istanbul University, Faculty of Pharmacy, Dept of Pharmaceutical Botany). This specimen has been stored at the Herbarium of Istanbul University (ISTE 98260).

*Preparation and GC/MS conditions for essential oil*

Essential oil was obtained using a Clevenger apparatus from the whole parts of plant, which was crumbled into small pieces and soaked in distilled water for 3 h. The obtained essential oil was dried over anhydrous Na_2_SO_4_ and stored at +4 °C for a sufficient period of time. The essential oil was diluted using CH_2_Cl_2 _(1:3 volume/volume) prior to GC/FID and GC/MS analysis. GC/FID performed using Thermo Electron Trace GC FID detector and GC/MS performed using same GC and Thermo Electron DSQ quadrupole for MS. 

The GC oven temperature was kept at 60 °C for 10 min and programmed to 280 °C at a rate of 4 °C/min and then kept constant at 280 °C for 10 min. A nonpolar Phenomenex DB5 fused silica column (30 m 0.32 mm, 0.25 μm film thickness) was used with helium at 1 mL/min (20 psi) as a carrier gas. The split ratio was adjusted to 1:50, the injection volume was 0.1 μL, and EI/MS was recorded at 70 eV ionization energy. The mass range was *m/z* 35–500 amu. Alkanes (C8-C24) were used as reference points in the calculation of Kovats Indices (KI) by the same conditions (12, 13).

Identification of the compounds was based on comparing their retention times and mass spectra with those obtained from authentic samples and/or the NIST and Wiley spectra as well as data from the published literature. GC/FID and GC/MS were replicated three times. (Mean RSD % < 0.1).

*Esterification of total fatty acids with* *GC/MS conditions*

A hundred milligram of the petroleum ether extract was refluxed in 0.1 M NaOH solution in 2 mL of methanol during 1 h, the solution was cooled and 5 mL of water was added. The aqueous mixture was neutralized with 0.5 mL of HCl solution, it was extracted with diethyl ether: hexane (3.5: 1: 1 mL). The separating organic phase was washed with 10 mL water, and dried over anhydrous Na_2_SO_4_. The solvent was evaporated in vacuum and then fatty acid methyl esters were obtained (14). The analyses was performed using a Thermo Scientific Polaris Q GC-MS/MS. GC/MS procedure described by Sabudak et al. was applied (14).

*Preparation of plant extracts*

Whole plants of *T. angustifolium* var. *angustifolium* (100 g) were dried, powdered, and then sequentially macerated with petroleum ether, acetone, methanol, and water for 24 h at 25 °C. After filtration, the solvents were evaporated to obtain crude extracts. This yielded 0.67% petroleum ether extract, 0.70% acetone extract, 4.5% methanol extract, and 2.8% water extract (w/w).

*Determination of total phenolic and flavonoid contents of extracts*

The amounts of phenolic and flavonoid contents in the crude extracts were expressed as pyrocatechol and quercetin equivalents, and they were calculated according to the following equations (15, 16):

Absorbance = 0.0128 pyrocatechol (μg) + 0.0324 (R^2 ^= 0.9920)

Absorbance = 0.1701 quercetin (μg) – 0.7078 (R^2 ^= 0.9939)

*Antioxidant activity* *of extracts*

*-Carotene bleaching method*

0.5 mg of  -carotene in 1 mL of chloroform was added into linoleic acid (25  L) and Tween 40 emulsifier (200 mg) mixture. After evaporating chloroform, 100 mL of distilled water saturated with oxygen was added followed by shaking, 160 μL of this mixture was transferred into different test tubes containing 40 μL of the sample solutions at different concentrations. The emulsion was added to each tube, the zero time absorbances of the values were read at 470 nm. The mixture was incubated for 2 h at 50 ^0^C (17, 18).

*Free radical scavenging activity method*

0.1 mM, 160 µL of DPPH solution in methanol was added to 40 µL of sample solutions in methanol at different concentrations. After 30 min. the absorbance values were read at 517 nm. The DPPH free radical scavenging potential was calculated using the following equation (18, 19):

DPPH scavenging effect Inhibition %=Acontrol-AsampleAcontrol×100

*A*_Control_ is the initial concentration of the DPPH^• ^

*A*_Sample_ is the absorbance of the remaining concentration of DPPH^•^ in the presence of the extracts or positive controls.

*ABTS* *cation radical decolorization assay*

 Seven milimolar ABTS in H_2_O was added to 2.45 mM potassium persulfate to produce ABTS^•+^ and solution was stored in the dark at 25 ºC for 12 h. The prepared solution was diluted with ethanol to get an absorbance of 0.700 ± 0.025 at 734 nm. ABTS^•+^ solution (160 µL) was added to each sample solution at different concentrations. After 30 min, the percentage inhibition at 734 nm was read for each concentration relative to a blank absorbance (methanol). The following equation was used to calculate the scavenging capability of ABTS^•+ ^(20):

$\mathrm{ABTS}•+\mathrm{scavenging effect}(\mathrm{Inhibition \%})=\frac{A_{\mathrm{control}}-A_{\mathrm{sample}}}{A_{\mathrm{control}}}\times 100$

*Cupric reducing antioxidant capacity (CUPRAC) method*

The petroleum ether and acetone extracts were dissolved in methanol, and methanol and water extracts in distilled water to prepare their stock solution at 1000 μg/mL concentration. Aliquots of 61 mL of 1.0 × 10^−2^ M copper (II) chloride, 61 μL of NH_4_OAc buffer (1 M, pH 7.0), and 61 μL of 7.5 × 10^−3^ M neocuproine solution were mixed, *x *μL sample solution (2.5, 6.25, 12.5, and 25 μL) and (67 − *x*) μL distilled water were added to make the final volume 250 μL. The tubes were stopped, and after 1 h, the absorbance at 450 nm was measured against a reagent blank (21).

*Anticholinesterase activity of the extracts*

All samples were dissolved in ethanol to prepare their stock solution at 4000 μg/mL concentration. Aliquots of 150 µL of 100 mM sodium phosphate buffer (pH 8.0), 10 μL of sample solution and 20 μL BChE (or AChE) solution were mixed and incubated for 15 min at 25 ºC, and DTNB (10 μL) is added. The reaction was then initiated by the addition of butyrylthiocholine iodide (or acetylthiocholine iodide) (10 μL). Final concentration of the tested solutions was 200 μg/mL (22). The hydrolysis of these substrates were monitored using a BioTek Power Wave XS at 412 nm. 

*Antimicrobial activity of extracts*

Five different microorganisms including Gram-positive bacteria (*Streptococcus pyogenes *ATCC19615 and *Staphylococcus aureus* ATCC 25923), Gram-negative bacteria (*Pseudomonas aeruginosa* ATCC 27853, *Escherichia coli* ATCC 25922), and yeast (*Candida albicans *ATCC10231) were purchased from Refik Saydam Sanitation Center (Turkey) and were used for detecting the antimicrobial activity of the samples. The disc diffusion method was employed for this purpose (23). Imipenem and nystatin were used as positive controls for bacteria and yeast, respectively.

*Statistical analysis*

The results of the antioxidant and anticholinesterase activity assays are expressed as the mean ± SD of three parallel measurements. The statistical significance was estimated using a Student’s t-test, where p-values *< *0.05 were considered significant.

## Results and Discussion

The essential oil composition of *T. angustifolium* var. *angustifolium* was determined by GC/MS analysis. As seen in Table 1, 21 components were determined, constituting 99.4% of the essential oil. The major components were hexatriacontene (23.0%), arachidic acid (15.5%) and *α*-selinene (10.0%). Some previous studies have investigated the essential oil composition of *Trifolium* species. According to Tajbakhsh *et al*. (24), the main constituents of the essential oil of *T. mazanderanicum* are tymol (41.3%), 8-cedren-13-ol-acetate (40.9%) and p-cymen-8-ol (5.3%).

**Table 1:** Chemical composition of the essential oil.

RI^a^	Rt (min)^b^	Constituents^c^	% Composition
1480	30.34	τ-Muurolene	1.5
1484	30.42	Valencene	1.6
1498	30.86	α-Selinene	10.0
1505	31.02	β-Himachalene	1.8
1746	35.52	2-Methyl heptadecane	1.9
1778	36.14	Pentadecanol	1.6
1800	36.45	Octadecane	2.2
1890	36.74	2-Methyl-1-hexadecanol	1.8
2185	38.34	Z-8-Octadecen-1-ol acetate	4.2
2171	38.98	Butyl phthalate	2.3
2109	40.01	Heneicosane	2.4
2259	40.13	2,5-di-tert octyl-p-benzoquinone	5.7
2366	40.59	Arachidic acid	15.5
1986	40.66	Hexadecanoic acid	6.3
2700	43.30	Heptacosane	1.5
2852	43.64	1-hexacosanol	4.2
2900	44.10	Nonacosane	2.9
3094	44.41	Ethyl iso-allocholate	2.9
3508	45.11	17-pentatriacontene	1.6
3600	46.50	Hexatriacontene	23.0
4400	47.12	Tetratetracontene	4.5
		Total	99.4

a  Retention indices (DB-5 column)

b Retention time (as minutes).

c Compounds listed in order of elution from a HP-5 MS column. A nonpolar Phenomenex DB-5 fused silica column

The fatty acid composition of the petroleum ether extract was determined by GC/MS analysis. As seen in Table 2, 12 components were identified, constituting 100% of the extract. The main constituents were palmitic acid (29.8%), linoleic acid (18.6%) and oleic acid (10.5%). Some previous studies have investigated the fatty acid composition of *Trifolium* species. According to Sabudak *et al*. (14), *T. stellatum *L., *T. Constantinopolitanum*, *T. nigrescens *subs. *petrisavii*, *T. balansae *Boiss. and *T. resupinatum *var. *resupinatum* have been investigated contents of fatty acids and lipids of using GC-MS. The five *Trifolium *species oils showed similar fatty acid profile. According to the GC-MS analysis, linolenic acid (16.6–31.1%, between), linoleic acid (5–11.3%) and palmitic acid (11.1–18.3%) were the major fatty acids. 

**Table 2:** GC/MS analysis of the petroleum ether extract

Rt (min)^a^	Constituents^b^	% Composition
12.00	Lauric acid	3.1
14.39	10-Undecenoic acid	1.3
18.60	Myristic acid	2.3
25.27	Palmitic acid	29.8
29.75	Phytol	2.6
30.64	Linoleic acid	18.6
30.77	Oleic acid	10.5
30.86	Linolenic acid	8.0
31.54	Stearic acid	5.2
37.38	Arachidic acid	5.4
39.36	Docosane	10.4
43.82	Behenic acid	2.8
	Total	100.0

a Retention time (as minutes).

b Compounds listed in order of elution from a HP-5 MS column. A nonpolar Phenomenex DB-5 fused silica colum.

The antioxidant activity of each extract was investigated using *β*-carotene bleaching, DPPH free radical scavenging, CUPRAC, and ABTS cation radical decolorization assays on their total phenolic and flavonoid contents. As shown in Table 3, total phenolic contents of all extracts were found to be higher than their flavonoid contents. The phenolic components of the petroleum ether extract was identified to be the richest (123.44 μg PEs/mg extract). The flavonoid components of the acetone extract was identified to be the richest. Sabudak *et al*. find out 66.10 to 82.71 µg PEs/mg extract phenolic contents of hexane extracts of five *Trifolium* species. Generally phenolic contents of polar extract are more than apolar extract in literature survey (25), otherwise there are some researches for contrary situations (26). According to report of Dahech *et al*. (26), they determined phenolic contents of hexane and butanol extracts of *Lycium shawii* fruits as 120 ve 75 mg GAE/g extract, respectively. Khanum *et al*. (27) reported that they find out very high amount of phenolic contents of nonpolar hexane extract of *Argyrolobium roseum* (792.5 +/- 0.55 gallic acid equivalent mg/g). Due to the fact that total phenolic content of nonpolar petroleum ether extract is more than total phenolic content of polar methanol extract, it can be related that phenolic contents of *Trifolium* species may have nonpolar character. 

**Table 3:** Total phenolic and flavonoid contents of the extracts^a^

**Extracts**	** Phenolic content** ** (μg PEs/mg extract)** b		**Flavonoid content** ** (μg QEs/mg extract)**^c^
Petroleum ether extract		123.44 ± 5.52	50.27 ± 0.58
Acetone extract		113.67 ± 2.76	65.03 ± 0.63
Methanol extract		71.35 ± 2.26	48.49 ± 0.31
Water extract		95.95 ± 2.21	49.12 ± 0.39

a Values expressed are means ± S.D. of three parallel measurements (*p *< 0.05)

b PEs, pyrocatechol equivalents (y=0.0128x + 0.0324 R^2^=0.9920)

c QEs, quercetin equivalents (y=0.1701x – 0.7078 R^2^=0.9939)

**Figure 1:**
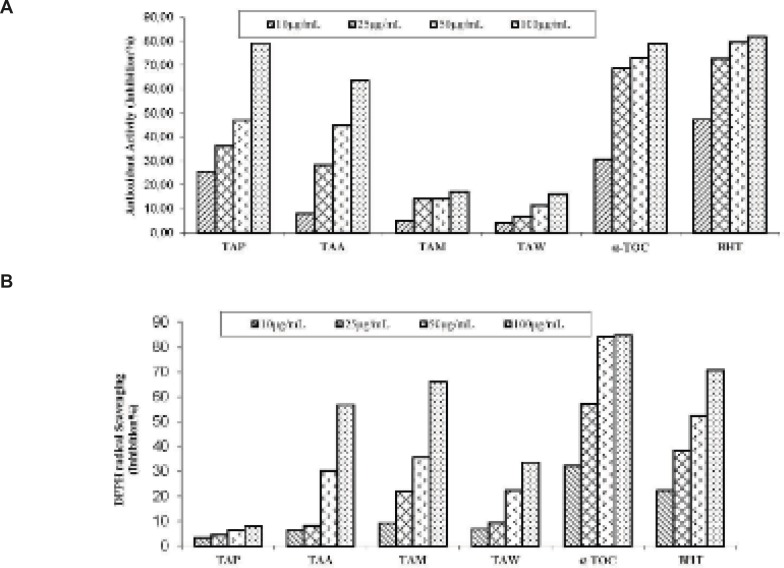
A: Inhibition (%) of lipid peroxidation; B: Inhibition (%) of DPPH free radical scavenging of the extracts, *α*-tocopherol and BHT. Values are means ± S.D., n=3, *p*<0.05, significantly different with Student’s t-test.

As shown in Figure 1A, the petroleum ether extract exhibited lipid peroxidation activity (47.12% and 79.12% inhibition) in the *β*-carotene bleaching method at concentrations of 50 and 100 μg/mL, respectively. The petroleum ether extract exhibited higher activity than *α*-tocopherol which was used as standard in the* β*-carotene bleaching method at concentration 100 μg/mL. Our results are in accordance with literature survey. According to Sabudak (13), the antioxidant potential of hexane extracts of *T. stellatum*, *T. balansae*, *T. nigrescens *subsp. *petrisavii*, *T. resupinatum *var. *resupinatum* and *T. constantinopolitanum *was determined by the DPPH free radical scavenging activity, *β*-carotene bleaching method and metal chelating activity. Sabudak *et al.* observed activity of nonpolar hexane extracts of five *Trifolium* species 56.59 to 82.88% in* β*-carotene bleaching assay at 50 μg/mL concentrations. 

As seen in Figure 1B, the acetone and methanol extracts exhibited 56.71% and 65.82% inhibition in the DPPH free radical scavenging activity assay method at 100 μg/mL, respectively. The petroleum ether and water extracts exhibited weak activity in the DPPH free radical scavenging activity assay method. Sabudak *et al.* also determined very weak activity of hexane extract of five *Trifolium *species in DPPH free radical scavenging activity assay (9.19 to 27.22% inhibition at 100 μg/mL). However Godavac *et al.* find out that methanol extract of *Trifolium pannonicum* has high activity in mentioned method (IC_50_ (μg/mL): 13.19). In this point of view our results are in accordance with literature survey. 

**Figure 2:**
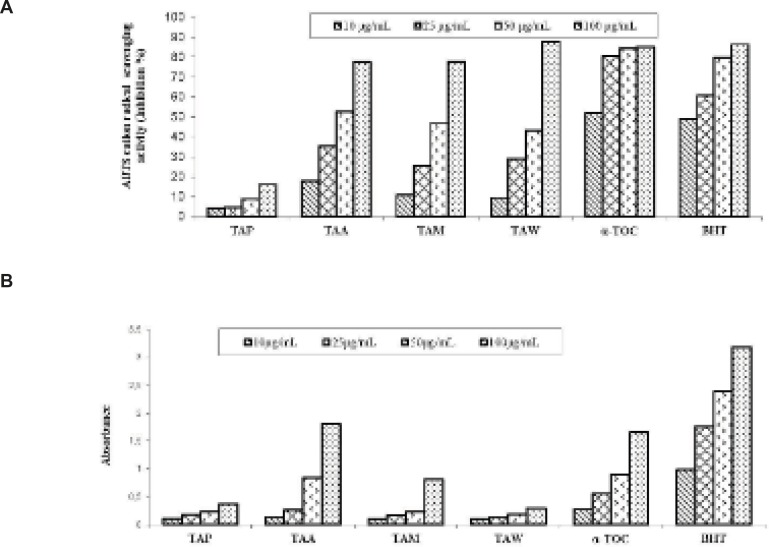
A: Inhibition (%) of ABTS cation radical scavenging; B: Cupric reducing antioxidant capacity of the extracts, *α*-tocopherol and BHT. Values are means ± S.D., n=3, *p*<0.05, significantly different with Student’s t-test.

As shown in Figure 2A, the water extract of *T. angustifolium* var. *angustifolium* exhibited 87.54% inhibition in the ABTS cation radical scavenging assay at 100 μg/mL. The water extract exhibited higher activity than *α*-tocopherol and BHT, which were used as standards in the ABTS cation radical scavenging assay at 100 μg/mL. As seen in Figure 2B, the acetone extract and *α-*tocopherol exhibited 1.80 and 1.65 absorbance in CUPRAC at 100 μg/mL, respectively. 

As shown in Table 4, the water extract indicated higher inhibitory effect against acetyl-cholinesterase enzyme (89.06%) than the reference compound, galanthamine, and the petroleum ether extract showed 57.60% inhibition activity against butyryl-cholinesterase enzyme, at 200 μg/mL. 

**Table 4:** Anticholinesterase activity of the extracts at 200 µg/mL^a^.

** Samples**	**Inhibition %** **against AChE**	**Inhibition %** **against BChE**
Petroleum ether extract	32.23±0.63	57.60±2.32
Acetone extract	54.14±2.32	47.40±0.23
Methanol extract	72.89±1.31	42.11±1.65
Water extract	89.06±2.32	32.09±0.13
Galanthamine^b^	88.86±0.76	82.20±0.35

a Values expressed are means ± S.D. of three parallel measurements (*p*<0.05)

b Standard drug

The antimicrobial activities of *T. angustifolium* var. *angustifolium* extracts against different microorganisms were assessed according to inhibition zone diameter. Results are presented in Table 5. The petroleum ether and water extracts showed no activity at all against the five tested microorganisms (Data not shown). The acetone and methanol extracts were active on all microorganisms tested with a small zone diameter indicating weak activity (inhibition zone << 12). 

**Table 5:** Zones of growth inhibition (mm) showing the antimicrobial activity of the extracts compared to that of positive controls.

Inhibition zone diameter	Microorganisms						
			*E.coli*	*S.aureus*	*S.pyogenes*	*P.aeruginosa*	*C.albicans*
	Acetone extract	10 mg/mL	10±0.2	9±0.2	11±0.3	9±0.6	10±0.4
		20 mg/mL	10±0.4	10±0.1	11±0.6	9±0.3	11±0.2
		30mg/mL	10±0.1	11±0.4	11±0.2	9±0.5	11±0.3
	Methanol extract	10 mg/mL	10±0.6	11±0.2	11±0.1	9±0.4	9±0.2
		20 mg/mL	11±0.4	11±0.5	12±0.1	10±0.1	10±0.3
		30 mg/mL	12±0.2	11±0.5	12±0.3	11±0.2	10±0.5
	IPM (10 μg/per disc)		19±1.4	39.5±0.7	27.5±0.7	12±0	-
	Nystatin (30 μg/per disc)		-	-	-	-	25±0.5

NZ: No zone

## Conclusions

The present study is the first fatty acid, essential oil and biological activity reports on *T. angustifolium* var. *angustifolium.* The water extract exhibited stronger ABTS cation radical scavenging activity than standard compounds,  -TOC and BHT. Additionally, the water extract indicated higher inhibitory effect against acetylcholinesterase (89.06%) than the reference compound, galanthamine. The water extract of *T. angustifolium* var. *angustifolium *can be investigated in terms of both phytochemical and biological aspects to find natural active compounds.

## References

[1] Zohary M, Trifolium L, Davis PH (1970). Flora of Turkey and the East Aegean Islands.

[2] Zohary M, Heler D (1984). The genus Trifolium.

[3] Baytop T (1984). Therapy with Medicinal Plants in Turkey.

[4] De Rijke E, Zafra Gomez A, Ariese Udo F, Brinkman TA, Gooljer C (2001). Determination of isoflavone glucoside malonates in Trifoliumpratense L. (red clover) extracts. J. Chromatogr. A.

[5] Oleszek W, Stochmal A (2002). Triterpene saponins and flavonoids in the seeds of Trifolium species. Phytochem.

[6] Mohamed KM, Ohtani K, Kasai R, Yamasaki K (1995). Oleanene glycosides from seeds of Trifolium alexandrinum. Phytochem.

[7] Mohamed KM, Mohamed HM, Ohtani K, Kasai R, Yamasaki K (1999). Megastigmane glycosides from seeds of Trifolium alexandrinum. Phytochem.

[8] Simonet AM, Stochmal A, Oleszek W, Macias FA (1999). Saponins and polar compounds from Trifolium resupinatum. Phytochem.

[9] Mohamed KH, Hassanean HA, Ohtani K, Kasai R, Yamasaki K (2000). Chalcanol glucosides from seeds of Trifolium alexandrinum. Phytochem.

[10] Sabudak T, Khan MTH, Choudhary MI, Oksuz S (2006). Potent tyrosinase inhibitors from Trifolium balansae. Nat. Prod. Res.

[11] Choi JM, Yoon BS, Lee SK, Hwang JK, Ryang R (2007). Antioxidant properties of neohesperidin dihydrochalcone: Inhibition of hypochlorous acid-induced DNA strand breakage, protein degradation, and cell death. Biol. Pharm. Bul.

[12] Gören AC, Piozzi F, Akcicek E, Kilic T, Carikci S, Mozioglu E, Setzer WN (2011). Essential oil composition of twenty-two Stachys species (mountain tea) and their biological activities. Phytochem. Lett.

[13] Polatoglu K, Demirci B, Demirci F, Goren N, Baser KHC (2012). The essential oil composition of Tanacetum densum (Labill ) Heywood ssp eginense Heywood from Turkey.. Rec. Nat. Prod.

[14] Sabudak T, Ozturk M, Goren AC, Kolak U, Topcu G (2009). Fatty acids and other lipid composition of five Trifolium species with antioxidant activity. Pharm. Biol.

[15] Slinkard K, Singleton VL (1977). Total phenol analyses: Automation and comparison with manual methods. Am. J. Enol. Viticult.

[16] Moreno MIN, Isla MI, Sampietro AR, Vattuone MA (2000). Comparison of the free radical-scavenging activity of propolis from several regions of Argentina. J. Ethnopharmacol.

[17] Miller HE (1971). A simplified method for the evaluation of antioxidants. J. Am. Oil Chem. Soc.

[18] Kosanic M, Rankovic B, Dasic M (2012). Mushrooms as possible antioxidant and antimicrobial agents. Iran. J. Pharm. Res.

[19] Blois MS (1958). Antioxidant determinations by the use of a stable free radical. Nature.

[20] Re R, Pellegrini N, Proteggente A, Pannala A, Yang M, Rice-Evans C (1999). Antioxidant activity applying an improved ABTS radical cation decolorization assay. Free Rad. Bio. Med.

[21] Apak R, Güçlü K, Özyürek M, Karademir SE (2004). Novel total antioxidant capacity index for dietary polyphenols and vitamins C and E, using their cupric ion reducing capability in the presence of neocuproine: CUPRAC Method. J. Agric. Food Chem.

[22] Ellman GL, Courtney KD, Andres V, Featherstone RM (1961). A new and rapid colorimetric determination of acetylcholinesterase activity. Biochem. Pharmacol.

[23] NCCLS (National Committee for Clinical Laboratory Standards) (1997). Performance Standards for Antimicrobial Disk Susceptibility Test.

[24] Tajbakhsh M, Khalilzadeh MA, Dabiri HA (2008). Volatile constituents of Lathyrus rotundifolius Willd and Trifolium mazanderanicum Rech. f. two Papilionaceae herbs growing wild in Iran.. J. Essent. Oil Res.

[25] Wang C, Yuan H, Bao X, Lan M (2013). In-vitro antioxidant and cytotoxic properties of ethanol extract of Alpinia oxyphylla. Pharm. Biol.

[26] Dahech I, Farah W, Trigui M, Hssouna AB, Belghith H, Belgith KS, Abdallah FB (2013). Antioxidant and antimicrobial activities of Lycium shawii fruits extract. Int. J. Biol. Macromol.

[27] Khanum R, Jahangir M, Aziz-ur-Rehman, Abbasi MA, Mazhar F, Kausar S, Riaz T, Ajaib M (2013). Phytochemical Screening and Antioxidant Evaluations of Different Fractions of Argyrolobium roseum. Asian J. Chem.

[28] Godevac D, Zdunic G, Savikin K, Vajs V, Menkovic N (2008). Antioxidant activity of nine Fabaceae species growing. Fitoterapia.

